# Neuropsychological correlates of reduced self-awareness of functional competency in persons with subjective memory complaints, mild cognitive impairment, and early probable Alzheimer’s dementia

**DOI:** 10.3389/fneur.2025.1666039

**Published:** 2025-10-06

**Authors:** Katie Coddaire, Molly McElvogue, Ashley M. Stokes, George P. Prigatano

**Affiliations:** ^1^Department of Clinical Neuropsychology, Barrow Neurological Institute, Phoenix, AZ, United States; ^2^The Barrow Neuroimaging Innovation Center, Barrow Neurological Institute, Phoenix, AZ, United States

**Keywords:** subjective memory complaints, mild cognitive impairment, early dementia, anosognosia, denial, reduced self-awareness, cognitive affective, motor correlates

## Abstract

Older adults with subjective memory complaints (SMC) often underestimate their cognitive and related functional competencies, while patients with mild cognitive impairment (MCI) or early probable Alzheimer’s disease dementia (AD) often overestimate their cognitive and functional abilities. We predicted that both cognitive (i.e., executive and memory) and non-cognitive (i.e., affective and motor) test performance of patients would be associated with reduced awareness of their functional limitations. Ten participants with SMC, 16 with MCI, and 10 with probable early AD were compared on measures of self and relatives’ perceptions of their daily functional capacities. Reduced self-awareness was behaviorally assessed by subtracting the patient’s subjective ratings of their functional abilities from the relatives’ (or significant others’) ratings of their functional abilities. Reduced self-awareness of functional competencies correlated with measures of language and calculation skills, memory, affect perception and expression, finger tapping movements, and overall cognitive status. The tendency to overestimate functional competences was associated with greater cognitive, affective, and motor impairments.

## 1 Introduction

Anosognosia is a historical term introduced by Babinski (1) to describe the clinical phenomenon in which patients appeared to lack “subjective” or conscious awareness of their paralyzed limb following a cerebrovascular accident (CVA). The term implied a complete loss or disruption of self-awareness of an impaired neurological function, which is often resolved within hours to days following the abrupt onset of a brain lesion. Progressively, it became obvious to clinicians that patients with various brain disorders tended to underreport their symptoms, including cognitive decline in dementia (2), socially inappropriate behaviors in post-acute patients with a history of severe traumatic brain injury (3), and movement disturbances in such conditions as Parkinson’s disease (4) and Huntington’s disease (5). It was also noted that an underreporting or apparent lack of awareness could exist for changes in personality associated with neurodegenerative diseases (6) and schizophrenia (7).

In these later conditions, the degree of “underreporting” varies in individuals, suggesting that there is a “gradient of subjective unawareness” that could not be captured in the historical term “anosognosia.” Consequently, the term “impaired self-awareness” (ISA) eventually emerged and has been used to describe “partial, but not complete unawareness” of cognitive, affective, or motor disturbances/impairments (8, 9).

Attempts to identify neuropsychological correlates of ISA have typically focused only on cognitive measures. More specifically, impaired performance on measures of executive functions and/or performance on various memory tests has been observed in patients with mild cognitive impairment (MCI) or early probable Alzheimer’s dementia (AD) (10–12). While this approach reflects the predominant cognitive profile of AD, it may provide incomplete insight into the underlying mechanisms. Additionally, this narrow focus overlooks the broader clinical implications: impaired self-awareness can compromise treatment adherence, hinder rehabilitation, and increase caregiver burden. Previous studies have also shown that patients with MCI and early probable AD may present with motor abnormalities (13–15) as well as various neuropsychiatric symptoms, which include apathy, indifference, or appearing “emotionally flat” (16–18). These non-cognitive features may also contribute to ISA. Investigating ISA across cognitive, motor, and affective domains offers a more comprehensive perspective and may reveal novel mechanisms that influence functional outcomes and quality of life throughout the Alzheimer’s disease continuum.

More recent studies have reported finger tapping abnormalities in MCI and early AD (15, 19–21). Finger tapping variability has been shown to be larger in the non-dominant hand in persons with MCI and AD compared to normal functioning individuals (19) and those with subjective memory complaints (SMC) (20). Furthermore, the number of “invalid” tapping responses (i.e., finger tapping movements that did not advance the number on a mechanical counter) was significantly greater in MCI and early probable AD patients versus those with SMC and normal controls (20).

Recent studies have shown that MCI patients tend to overestimate their performance across cognitive domains (22), especially regarding visuospatial memory (23). However, individuals with SMC tend to overreport (not underreport) their memory difficulties (24, 25). This pattern may reflect *reduced awareness of their abilities versus any disabilities or impairments* observed on clinical examination. Empirical studies examining the neuropsychological correlates of ISA in persons with MCI and AD have typically excluded individuals with SMC. Including this group in studies of ISA may clarify what factors influence disparities in the perception of functional abilities by the patient and an informant.

Clinically, ISA can be measured using the Patient Competency Rating Scale (PCRS) and PCRS-Relative Form (PCRS-R) (26). The PCRS is a brief questionnaire consisting of a series of statements that assess a patient’s perceived functional competency across various domains, such as activities of daily living, interpersonal and social functioning, cognitive abilities, and emotional and behavioral regulation. The parallel form, the PCRS-R, is completed by a relative or “significant other” (friend, partner, etc.) who knows the patient well. All responses are rated on a Likert scale, ranging from one (Not at all capable) to five (Very capable). By analyzing the discrepancy between patient and informant ratings, these two measures have been used in several studies to measure ISA, including in traumatic brain injury (27, 28); MCI and early probable AD (29); Parkinson’s disease (30); multiple sclerosis (31, 32); brain tumors and other cancers (33); and stroke (34). In patients with known brain disorders, ISA broadly refers to a disturbance in self-awareness that can affect multiple domains, including cognitive, emotional, and functional abilities. For the purposes of the present study, we operationalize ISA as the degree to which patients either overestimate or underestimate their functional autonomy compared to informant reports of everyday competence.

While the reliability and validity of relatives’ reports has been questioned, several studies have shown that their ratings are typically positively associated with the patient’s actual neuropsychological test performance (35–38). This has been found in both normally functioning individuals (39) and those with cognitive impairments (40, 41).

As part of an ongoing study in persons with SMC, MCI, and early probable AD (20), patients and their accompanying relative were asked to complete the PCRS and PCRS-R, respectively. Based on the existing literature, we predicted that people with MCI and probable early AD would tend to report higher levels of functional abilities on the PCRS compared to relatives’ reports on the PCRS-R. Moreover, we predicted this tendency to describe oneself as being more functionally competent than what relatives report would be significantly associated with worse test performance on measures of executive functioning and memory, as well as on measures of affect expression and perception. We further hypothesized, given recent research findings (20), that the number of invalid tapping responses (particularly in the non-dominant hand) using the modified version of the Halstead Finger Tapping Test (42) would significantly correlate with the degree of reduced awareness (i.e., overreporting or overestimating one’s functional abilities). Finally, it was predicted that people with SMC would tend to report lower levels of functional abilities on the PCRS compared to relatives’ reports of their functional abilities on the PCRS-R.

## 2 Materials and methods

### 2.1 Subjects

This study is part of a larger ongoing study on the motor correlates of finger tapping behaviors in older adults with known or subjectively reported memory impairments (20). Participants were initially referred for a clinical neuropsychological evaluation due to concerns about cognitive impairment raised by themselves, their family members, or their physicians.

As part of their clinical evaluation, each participant was administered a comprehensive battery of neuropsychological tests by a single clinical neuropsychologist (G. P. P., >40 years of expertise). If their neuropsychological test performance indicated SMC, MCI of the amnestic type, or probable early dementia of the Alzheimer’s type, research staff obtained informed consent in accordance with the Institutional Research Board (IRB) of Saint Joseph’s Hospital and Medical Center and the Barrow Neurological Institute (BNI) in Phoenix, Arizona, United States.

Based on a clinical consensus between a geriatric psychiatrist (>20 years’ experience) and a clinical neuropsychologist (G. P. P., >50 years’ experience), three clinical groups were identified. Participants with SMC reported memory issues but obtained a standard score of at least 90 for the Wechsler Adult Intelligence Scale - Fourth Edition (WAIS-IV) (43) Full-Scale Intelligence Quotient (FSIQ) and within 1.5 standard deviations of the mean on two memory tests. Those classified as MCI met the Petersen 2004 criteria (44) with memory scores at least 1.5 standard deviations below the mean on two tests, while maintaining an FSIQ of at least 90. Although they had minor deficits in non-memory areas, they functioned independently in daily life. The third group scored at least 1.5 standard deviations below the mean in memory and intellectual tests, with FSIQ below 90, and showed significant impairments in at least two other cognitive domains (e.g., language and executive functions). They required assistance for daily activities, consistent with mild to moderate Alzheimer’s disease, per McKhann et al. criteria (45).

### 2.2 Selection criteria

Eligible participants provided informed consent, were between ages 60 and 84 years, and English was their primary language. Individuals were excluded if they declined to participate after receiving additional study details; if they had a neurological or psychiatric history that might affect neuropsychological performance (e.g., severe traumatic brain injury, brain tumor, cerebrovascular accident, multiple sclerosis, psychosis, or drug addiction); if they had a history of learning disability; or if neuropsychological, neurological, or neuroimaging findings suggested non-Alzheimer’s-related brain pathology (e.g., severe cerebrovascular disease with infarcts or extensive white matter hyperintensities, Lewy body dementia, frontotemporal dementia, or Parkinsonism).

Since the current study focused on measuring reduced self-awareness using the PCRS and PCRS-Relative forms, only participants who had both a self- and relative version of the PCRS completed were included in the present study. Given these selection criteria, the current study included a total of 36 participants, with 10 SMC, 16 MCI, and 10 early probable AD with mild to moderate dementia.

### 2.3 Procedures

#### 2.3.1 Neuropsychological measures

Handedness was assessed via self-report (right-handed, left-handed, or ambidextrous). Research participants were administered the following tests: the Wechsler Adult Intelligence Scale-Fourth Edition (WAIS-IV) (43), which provides a Verbal Comprehension Index (VCI), Perceptual Reasoning Index (PRI), Working Memory Index (WMI), and Processing Speed Index (PSI), as well as a measure of Full-Scale Intelligence Quotient (FSIQ); the Barrow Neurological Institute Screen for Higher Cerebral Functions (BNIS) (46), which includes seven subtest scores: Speech and Language, Orientation, Attention and Concentration, Visuospatial and Problem Solving, Memory, Affect, Awareness vs. Performance, as well as a Total Score; the Rey Auditory Verbal Learning Test (RAVLT) (47); the Brief Visual Memory Test - Revised (BVMT-R) (48); the Trail Making Test, Parts A and B (TMT) (49); and the modified version of the Halstead Finger Tapping Test (HFTT) (42). This latter test measures speed of finger tapping, variability of finger tapping speeds, and the number invalid tapping responses in both hands when performing this task [see (20) for details concerning this methodology].

#### 2.3.2 Subjective perceptions/experiences of functional competency measures and the measure of reduced self-awareness

The PCRS (26) was administered to each patient to evaluate their subjective perceptions of their functional abilities in everyday life. The PCRS is a 30-item questionnaire used to assess the person’s perception (or subjective experience) of their functional abilities across various domains, including activities of daily living, behavior and emotion, cognition, and physical function. Scores range from 30 (meaning an individual perceives they cannot perform any of the tasks described), to 150 (meaning the individual perceives all tasks “can be done with ease”).

The PCRS-R is the parallel form given to the relative to complete and contains the same questions but requires the relative to rate the patient’s level of competency on each of the questions from their point of view or observations. Again, scores can range from 30 to 150 points.

To quantify reduced awareness, a discrepancy score was calculated by subtracting the PCRS Total score from the PCRS-R Total score. Positive values indicate that the patient rated their functional abilities as greater than the relative did (i.e., overestimated functional ability), while negative values indicate the patient rated themselves as more impaired than the relative (i.e., underestimated functional ability).

### 2.4 Statistical analyses

All analyses were performed using a custom script developed in R (Version 4.3.2) and RStudio (Version 2023.12.1 + 402). Analyses of demographic variables across the three participant groups were conducted using one-way analysis of variance (ANOVA) for age and education level and Pearson chi-square tests for sex and handedness. All neuropsychological test scores were normed according to age and education level, when applicable, and ANOVAs were used for analysis of psychometric test findings across groups. Effect sizes obtained using ANOVAs were calculated using eta squared values (η^2^). Spearman’s rank-order correlation (or point biserial correlation) coefficients were computed to assess relationships among the PCRS Total Scores, the PCRS-R Total Scores, and the PCRS Discrepancy Scores with demographic characteristics and neuropsychological test scores. While multiple correlations were calculated, significant group differences were set at the *p* < 0.05 level given the specific *a priori* predictions made regarding expected correlational findings. Both forward and backward hierarchical multiple regression analyses were calculated to examine predictors of the PCRS discrepancy scores. Because these analyses were exploratory and intended to generate hypotheses, we reported uncorrected *p*-values and comparisons that remained significant after False Discovery Rate (FDR) correction.

## 3 Results

### 3.1 Demographic results

There were no statistically significant differences among groups in terms of ratio of males to females (*p* = 0.56), age (*p* = 0.16), level of education (*p* = 0.78), or handedness (*p* = 0.21). Three of the seven early probable AD dementia participants were left-hand-dominant (42.8%) (Table 1). As a result, all HFTT data was analyzed using dominant hand (DH) versus non-dominant hand (NDH) instead of right-hand versus left-hand comparisons.

**Table 1:** Demographic data and psychometric test findings across groups.

Demographic data					
Group	SMC	MCI	AD	Group comparisons	*η^2^*
N (Females)	10 (7)	16 (8)	10 (5)	Pearson Chi-square: x2 = 1.17 (*p* = 0.56)	–
Age in Years (SD)	70.7 (6.5)	75.0 (5.1)	74.9 (6.3)	ANOVA: *F* = 1.93 (*p* = 0.16)	0.10
Education in Years (SD)	15.5 (2.1)	14.9 (3.0)	14.7 (2.5)	ANOVA: *F* = 0.25 (*p* = 0.78)	0.02
Handedness [R/L]	9/1	15/1	7/3	Pearson Chi-square: x2 = 3.10 (*p* = 0.21)	–

Psychometric test findings across groups					
Neuropsychological test score	SMC Mean (SD)	MCI Mean (SD)	AD Mean (SD)	Group comparisons ANOVA	*η^2^*
BNIS total raw score***	44.70 (3.06)	36.53 (4.09)	33.60 (3.37)	*F* = 25.69 (*p* < 0.001)	0.62
BNIS speech and lang.	15.00 (0.00)	14.00 (1.41)	13.90 (0.99)	*F* = 2.77 (*p* = 0.08)	0.17
BNIS orientation***	3.00 (0.00)	2.31 (0.75)	1.60 (0.84)	*F* = 9.32 (*p* < 0.001)	0.40
BNIS attn. and concent.	2.00 (0.76)	1.54 (0.66)	1.50 (0.71)	*F* = 1.39 (*p* = 0.27)	0.09
BNIS visuosp. and PS	5.88 (1.64)	4.77 (1.48)	4.40 (1.71)	*F* = 2.01 (*p* = 0.15)	0.13
BNIS memory***	5.63 (1.19)	1.85 (2.03)	0.40 (0.84)	*F* = 26.98 (*p* < 0.001)	0.66
BNIS affect	3.38 (1.06)	2.62 (0.96)	2.80 (0.63)	*F* = 1.82 (*p* = 0.18)	0.12
BNIS aware. and perform.***	0.88 (0.35)	0.23 (0.44)	0.00 (0.00)	*F* = 15.80 (*p* < 0.001)	0.53
WAIS-IV FSIQ***	111.9 (14.70)	101.25 (9.97)	84.9 (12.84)	*F* = 12.47 (*p* < 0.001)	0.43
WAIS-IV VCI**	113.30 (12.78)	101.94 (9.94)	93.3 (13.50)	*F* = 7.25 (*p* = 0.002)	0.31
WAIS-IV PRI***	111.40 (18.83)	99.19 (11.58)	84.1 (13.25)	*F* = 9.11 (*p* < 0.001)	0.36
WAIS-IV WMI*	106.50 (12.34)	99.63 (9.72)	89.6 (11.60)	*F* = 5.99 (*p* = 0.006)	0.27
WAIS-IV PSI**	108.4 (18.82)	104.44 (11.37)	83.7 (19.51)	*F* = 7.09 (*p* = 0.003)	0.30
RAVLT TR T-score***	56.90 (9.40)	36.69 (12.28)	25.5 (13.80)	*F* = 17.68 (*p* < 0.001)	0.52
RAVLT LD T-score***	57.80 (10.55)	28.13 (11.63)	21.90(10.62)	*F* = 31.26 (*p* < 0.001)	0.65
BVMT-R DR T-score***	49.80 (14.67)	25.31 (5.99)	23.30 (6.52)	*F* = 26.78 (*p* < 0.001)	0.62
Trails A T-score*	45.60 (9.83)	47.75 (11.33)	36.80 (9.92)	*F* = 3.45 (*p* = 0.04)	0.17
Trails B T-score***	52.90 (9.90)	47.31 (10.98)	31.57 (9.85)	*F* = 8.97 (*p* < 0.001)	0.37
HFTT DH speed	43.26 (6.33)	40.72 (5.29)	38.10 (5.35)	*F* = 2.12 (*p* = 0.14)	0.11
HFTT NDH speed*	38.84 (5.34)	35.01 (6.71)	30.58 (8.95)	*F* = 3.41 (*p* = 0.04)	0.17
HFTT DH range*	9.70 (5.56)	13.38 (7.14)	19.30 (8.18)	*F* = 4.73 (*p* = 0.02)	0.22
HFTT NDH range*	9.10 (4.25)	11.75 (3.75)	16.10 (7.22)	*F* = 4.92 (*p* = 0.01)	0.23
HFTT DH invalid taps**	0.30 (0.67)	1.50 (1.71)	3.10 (1.95)	*F* = 7.86 (*p* = 0.002)	0.32
HFTT NDH invalid taps**	0.90 (1.60)	3.56 (2.53)	4.80 (2.30)	*F* = 7.99 (*p* = 0.002)	0.33
HFTT total invalid taps***	1.20 (2.15)	5.06 (3.36)	7.90 (4.07)	*F* = 10.41 (*p* < 0.001)	0.39
PCRS-self total score	121.10 (16.70)	127.19 (8.80)	118.70 (8.38)	*F* = 1.92 (*p* = 0.16)	0.10
PCRS-relative total score**	126.90 (14.96)	116.44 (14.46)	103.60 (13.43)	*F* = 6.64 (*p* = 0.004)	0.29
PCRS discrepancy total score**	−5.80 (12.58)	10.75 (10.12)	15.10 (18.84)	*F* = 6.74 (*p* = 0.004)	0.29

*p ≤ 0.05. **p ≤ 0.005. ***p ≤ 0.001. SMC, subjective memory complaints; MCI, Mild Cognitive Impairment; AD, early probable Alzheimer’s disease with mild to moderate dementia; ANOVA, Analysis of Variance; SD, standard deviation; R/L, right/left; BNIS, Barrow Neurological Institute Screen for Higher Order Cerebral Functions; BNIS Speech and Lang., BNIS Speech and Language subscore; BNIS Attn. and Concent., BNIS Attention and Concentration subscore; BNIS Visuosp. and PS, BNIS Visuospatial and Problem Solving subscore; BNIS Aware. and Perform., BNIS Awareness and Performance subscore; WAIS-IV, Wechsler Adult Intelligence Scale, Fourth Edition; FSIQ, Full Scale Intelligence Quotient; VCI, Verbal Comprehension Index; PRI, Perceptual Reasoning Index; WMI, Working Memory Index; PSI, Processing Speed Index; RAVLT, Rey Auditory Verbal Learning Test; TR, Total Recall; LD, Long Delay; BVMT-R, Brief Visuospatial Memory Test-Revised; DR, Delayed Recall; Halstead Finger Tapping Test (HFTT) DH, dominant hand; NDH, non-dominant hand; PCRS, Patient Competency Rating Scale.

### 3.2 Psychometric test results

Given the diagnostic criteria for each of the three patient groups, there were expected significant differences in performance levels found on cognitive and motor measures across the SMC, MCI, and AD groups (Table 1). Groups differed on the BNIS Total Raw Score [*F*(2, 32) = 25.69, *p* < 0.001, η^2^ = 0.62], with the SMC group achieving the highest scores [mean ± standard deviation (SD) = 44.70 ± 3.06], followed by the MCI (36.53 ± 4.09) and AD groups (33.60 ± 3.37); the BNIS Orientation subtest score [*F*(2, 28) = 9.32, *p* < 0.001, η^2^ = 0.40], with the SMC group scoring perfectly (3.00 ± 0.00), while the MCI and AD groups showed lower scores (2.31 ± 0.75 and 1.60 ± 0.84, respectively); the BNIS Memory subtest score [*F*(2, 28) = 26.98, *p* < 0.001, η^2^ = 0.66]; and the BNIS Awareness and Performance subtest score, with the AD group exhibiting the lowest scores [*F*(2, 28) = 15.80, *p* < 0.001, η^2^ = 0.53].

Groups significantly differed on each of the Wechsler Scale Composite scores and the Full-Scale IQ scores. FSIQ scores were significantly different [*F*(2, 33) = 12.47, *p* < 0.001, η^2^ = 0.43], with the SMC group exhibiting the highest composite IQ scores (111.9 ± 14.70), followed by the MCI (101.25 ± 9.97) and AD groups (84.9 ± 12.84). Significant group differences were also observed in the Verbal Comprehension Index [*F*(2, 33) = 7.25, *p* = 0.002, η^2^ = 0.31], Perceptual Reasoning Index [*F*(2, 33) = 9.11, *p* < 0.001, η^2^ = 0.36], Working Memory Index [*F*(2, 33) = 5.99, *p* = 0.006, η^2^ = 0.27], and Processing Speed Index [*F*(2, 33) = 7.09, *p* = 0.003, η^2^ = 0.30].

The RAVLT Total Recall T-scores demonstrated significant differences across groups [*F*(2, 33) = 17.68, *p* < 0.001, η^2^ = 0.52], with the AD group exhibiting the expected lowest scores (25.5 ± 13.80). The RAVLT Long Delay T-scores were significantly different [*F*(2, 33) = 31.26, *p* < 0.001, η^2^ = 0.65], with the SMC group scoring highest (57.80 ± 10.55), indicating better memory retention compared to the MCI (28.13 ± 11.63) and AD groups (21.90 ± 10.62). The BVMT-R Delayed Recall T-scores also showed significant differences [*F*(2, 33) = 26.78, *p* < 0.001, η^2^ = 0.62], with the SMC group having the highest score (49.80 ± 14.67).

Trails A and B T-scores revealed significant differences across groups. Trails A T-scores were significantly lower in the AD group [*F*(2, 33) = 3.45, *p* = 0.04, η^2^ = 0.17]. Trails B T-scores were significantly different between groups [*F*(2, 30) = 8.97, *p* < 0.001, η^2^ = 0.37]. Substantial impairments in the AD group (31.57 ± 9.85) compared to SMC (52.90 ± 9.90) and MCI groups (47.31 ± 10.98) were found.

Non-dominant hand speed was significantly slower in the AD group [*F*(2, 33) = 3.41, *p* = 0.04, η^2^ = 0.17], with the lowest mean compared to SMC and MCI. Both dominant hand range scores [*F*(2, 33) = 4.73, *p* = 0.02, η^2^ = 0.22] and non-dominant hand range scores [*F*(2, 33) = 4.92, *p* = 0.01, η^2^ = 0.23] indicated greater variability in the AD group. The number of invalid tapping responses was higher in the AD group, with dominant hand invalid taps [*F*(2, 33) = 7.86, *p* = 0.002, η^2^ = 0.32] and non-dominant hand invalid taps [*F*(2, 33) = 7.99, *p* = 0.002, η^2^ = 0.33] significantly elevated, as well as total invalid taps [*F*(2, 33) = 10.41, *p* < 0.001, η^2^ = 0.39].

### 3.3 Patients’ and relatives’ PCRS ratings

**Figure 1:**
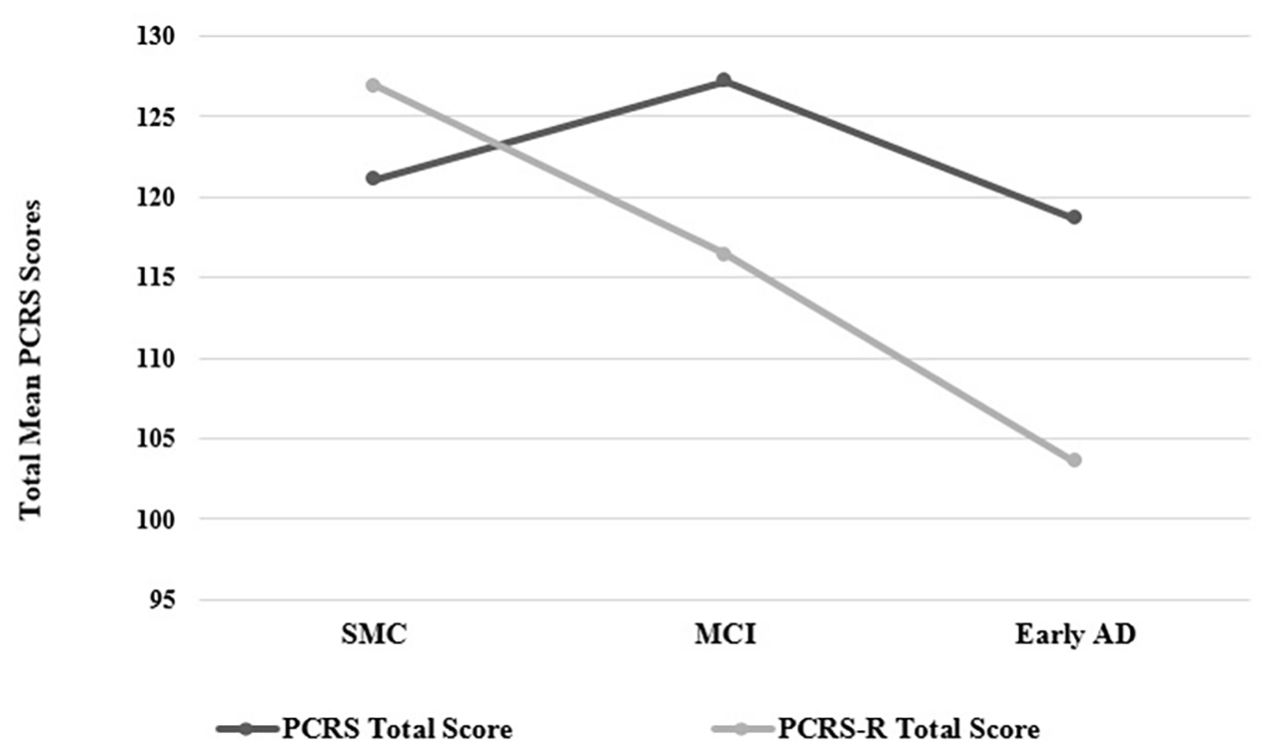
Plots of the PCRS and PCRS-R total scores by group (see Table 1 for mean and standard deviation values). PCRS, Patient Competency Rating Scale; PCRS-R, Patient Competency Rating Scale-Relative form; SMC, subjective memory complaint; MCI, Mild Cognitive Impairment; Early AD, early probable Alzheimer’s disease with mild to moderate dementia.

Patients’ self-reported ratings of competency (PCRS Total Scores) did not show significant variation across groups [*F*(2, 33) = 1.92, *p* = 0.16, *η^2^* = 0.10; Table 1]. However, relatives’ ratings of the patient competencies (PCRS-R Total Scores) did vary significantly across groups [*F*(2, 33) = 6.64, *p* = 0.004, *η^2^* = 0.29]. Relative ratings of the SMC participants described them as functionally more competent than those in the MCI and early probable AD dementia groups. Additionally, the discrepancy scores between the PCRS and PCRS-R revealed differences across groups [*F*(2, 33) = 6.74, *p* = 0.004, *η^2^* = 0.29], with SMC participants reporting less functional abilities than what their relatives reported about their functional abilities, while the MCI and early probable AD dementia participants reported greater levels of functional abilities than what their relatives reported regarding their daily competencies (Figure 1). These findings support two of the major hypotheses of this study.

#### 3.3.1 Patients’ self-reported functional competencies and their neuropsychological test performance

Correlational analyses showed no significant relationships (*p* < 0.05) between subjective self-reported functional competency (as measured by the PCRS Total score) and objective neuropsychological test scores (Table 2), including cognitive, motor, and affective functioning.

**Table 2:** Correlations of neuropsychological test performance with the PCRS-self and the PCRS-relative ratings.

	PCRS-self total score		PCRS-relative total score	
Neuropsychological test score	Spearman’s ρ	*p*	Spearman’s ρ	*p*
BNIS total raw score	0.17	0.34	0.58	<0.001***^+^
BNIS speech and language	0.02	0.93	0.61	<0.001***^+^
BNIS orientation	0.11	0.56	0.43	0.02*^+^
BNIS atten. and concent.	−0.15	0.41	0.08	0.68
BNIS Visuosp. and P. S.	0.25	0.18	0.39	0.03*^+^
BNIS memory	0.25	0.17	0.53	0.002**^+^
BNIS affect	0.17	0.37	0.51	0.003**^+^
BNIS aware. and perform.	0.19	0.30	0.40	0.03*^+^
WAIS-IV FSIQ	0.16	0.34	0.54	< 0.001***^+^
WAIS-IV VCI	0.11	0.52	0.47	0.004**^+^
WAIS-IV PRI	−0.07	0.69	0.41	0.01*^+^
WAIS-IV WMI	0.20	0.25	0.49	0.003**^+^
WAIS-IV PSI	0.28	0.10	0.38	0.02*^+^
RAVLT TR T-score	0.08	0.62	0.44	0.01*^+^
RAVLT LD T-score	0.04	0.84	0.40	0.02*^+^
BVMT-R DR T-score	0.02	0.91	0.54	<0.001***^+^
Trails A T-score	0.21	0.22	0.34	0.04*^+^
Trails B T-score	0.32	0.07	0.63	<0.001***^+^
HFTT DH speed	0.18	0.29	0.07	0.69
HFTT NDH speed	0.19	0.27	0.23	0.17
HFTT DH range	0.03	0.87	−0.23	0.17
HFTT NDH range	−0.12	0.50	−0.32	0.06
HFTT DH invalid taps	−0.14	0.41	−0.33	0.05
HFTT NDH invalid taps	−0.004	0.98	−0.37	0.02*
HFTT total invalid taps	−0.08	0.66	−0.42	0.01*

Uncorrected: *p ≤ 0.05, **p ≤ 0.005, ***p ≤ 0.001. ^+^Significant after FDR correction at *p* ≤ 0.05. PCRS, Patient Competency Rating Scale; BNIS, Barrow Neurological Institute Screen for Higher Order Cerebral Functions; BNIS Atten. and Concent., BNIS Attention and Concentration subscore; BNIS Visuosp. and P. S., BNIS Visuospatial and Problem Solving subscore; BNIS Aware. and Perform., BNIS Awareness and Performance subscore; WAIS-IV, Wechsler Adult Intelligence Scale, Fourth Edition; FSIQ, Full Scale Intelligence Quotient; VCI, Verbal Comprehension Index; PRI, Perceptual Reasoning Index; WMI, Working Memory Index; PSI, Processing Speed Index; RAVLT, Rey Auditory Verbal Learning Test; TR, Total Recall; LD, Long Delay; BVMT-R, Brief Visuospatial Memory Test-Revised; DR, Delayed Recall; Halstead Finger Tapping Test (HFTT); DH, dominant hand; NDH, non-dominant hand.

#### 3.3.2 Relatives’ ratings of the PCRS and the patients’ neuropsychological test performance

Several moderate to high correlations were found between the patients’ performance on neuropsychological tests and relatives’ reports of the patient’s functional competency as measured by the PCRS-R (Table 2). A clear pattern of findings emerged in which the relatives’ view of higher levels of functional competencies in everyday life was parallel with higher levels of performance on many neuropsychological measures.

The BNIS Total Raw Score (*ρ* = 0.58, *p* < 0.001), the BNIS Speech and Language subtest (*ρ* = 0.61, *p* < 0.001), the WAIS-IV FSIQ (*ρ* = 0.54, *p* < 0.001), the BVMT-R Delayed Recall T-score (*ρ* = 0.54, *p* < 0.001), and Trails B T-score (*ρ* = 0.63, *p* < 0.001) all were positively and strongly correlated with the total PCRS-R scores. Moderate negative correlations were observed with NDH Invalid Taps (*ρ* = −0.37, *p* = 0.02) and Total Invalid Taps (*ρ* = −0.42, *p* = 0.01) and the PCRS-R.

#### 3.3.3 Reduced self-awareness and neuropsychological test performance

Table 3 lists the correlations between the various neuropsychological test scores and the PCRS versus PCRS-R discrepancy scores. The BNIS Speech and Language subtest, which includes basic mathematical tasks, demonstrated a strong negative correlation (*ρ* = −0.65, *p* < 0.001). Greater overestimation of functional abilities was associated with poorer performance on speech, language and calculation tasks. While initial analyses indicated significant associations for the BNIS Orientation (*ρ* = −0.40, *p* = 0.03), Memory (*ρ* = −0.38, *p* = 0.03), and Affect subtests (*ρ* = −0.36, *p* = 0.047), these did not remain significant after applying the FDR correction. Lower BNIS Total score was also associated with greater overestimation of functional abilities (*ρ* = −0.48, *p* = 0.003).

**Table 3:** Correlations with PCRS discrepancy scores and neuropsychological test results.

	PCRS discrepancy scores	
Neuropsychological test score	Spearman’s ρ	*p*
BNIS total raw score	−0.48	0.003**^+^
BNIS speech and language	−0.65	<0.001***^+^
BNIS orientation	−0.40	0.03*
BNIS atten. and concentration	−0.22	0.24
BNIS visuosp. and prob. solv.	−0.14	0.45
BNIS memory	−0.38	0.03*
BNIS affect	−0.36	0.047*
BNIS aware. and perform.	−0.31	0.09
WAIS-IV FSIQ	−0.36	0.03*^+^
WAIS-IV VCI	−0.39	0.02*^+^
WAIS-IV PRI	−0.42	0.01*^+^
WAIS-IV WMI	−0.29	0.09
WAIS-IV PSI	−0.12	0.50
RAVLT total recall T-score	−0.41	0.01*^+^
RAVLT long delay T-score	−0.43	0.01*^+^
BVMT-R delayed recall T-score	−0.54	<0.001***^+^
Trails A T-score	−0.10	0.58
Trails B T-score	−0.30	0.08
HFTT DH speed	0.08	0.63
HFTT NDH speed	−0.17	0.33
HFTT DH range	0.19	0.27
HFTT NDH range	0.31	0.06
HFTT DH invalid taps	0.24	0.16
HFTT NDH invalid taps	0.44	0.01*^+^
HFTT total invalid taps	0.41	0.01*^+^

Uncorrected: *p ≤ 0.05, **p ≤ 0.005, ***p ≤ 0.001. ^+^Significant after FDR correction at *p* ≤ 0.05. PCRS, Patient Competency Rating Scale; BNIS, Barrow Neurological Institute Screen for Higher Order Cerebral Functions; BNIS Attent. and Concentration, BNIS Attention and Concentration subscore; BNIS Visuosp. and Prob. Solv., BNIS Visuospatial and Problem Solving subscore; BNIS Aware. and Perform., BNIS Awareness and Performance subscore; WAIS-IV, Wechsler Adult Intelligence Scale, Fourth Edition; FSIQ, Full Scale Intelligence Quotient; VCI, Verbal Comprehension Index; PRI, Perceptual Reasoning Index; WMI, Working Memory Index; PSI, Processing Speed Index; RAVLT, Rey Auditory Verbal Learning Test; BVMT-R, Brief Visuospatial Memory Test-Revised; Halstead Finger Tapping Test (HFTT); DH, dominant hand; NDH, non-dominant hand.

The WAIS-IV FSIQ (*ρ* = −0.36, *p* = 0.03), Verbal Comprehension Index (*ρ* = −0.39, *p* = 0.02), and Perceptual Reasoning Index (*ρ* = −0.42, *p* = 0.01) showed mild to moderate negative correlations with this discrepancy measure. Lower performance levels of the RAVLT and BVMT-R Delayed Recall measures were associated with higher overestimation scores (RAVLT Long Delay T-score: *ρ* = −0.43, *p* = 0.01; BVMT-R Delayed Recall T-score: ρ = −0.54, *p* < 0.001).

Finally, both a greater number of NDH invalid tapping movements and the total number of invalid tapping movements were moderately and positively correlated with the patient’s tendency to describe themselves as more competent than relatives’ ratings of them (*ρ* = 0.44, *p* = 0.01, and *ρ* = 0.41, *p* = 0.01, respectively).

Collectively, these findings support the third hypothesis that both cognitive and non-cognitive neuropsychological functioning were associated with reduced awareness of one’s actual functioning.

### 3.4 Predicting ISA: hierarchical multiple regression analyses

While several variables correlated with discrepancy scores, the four variables that exhibited the strongest correlations were the BNIS Speech and Language subtest score, the BVMT-R Delayed recall T-score; the number of NDH invalid taps, and the BNIS Total Score. These scores were entered into a forward hierarchical multiple regression model to predict the discrepancy scores between the PCRS and PCRS-R.

The BNIS Speech and Language subtest score alone accounted for 30% of the variance in the discrepancy scores [*R*^2^ = 0.30, *F*(1, 29) = 12.67, *p* = 0.001]. The addition of the BVMT-R Delayed Recall T-score further increased the explained variance to 42% [*R*^2^ = 0.42, *F*(2, 28) = 10.27], with a significant change [Δ*F* = 5.41, *p* = 0.03]. However, subsequent inclusion of NDH Invalid Taps and BNIS Total Raw Score did not significantly enhance the model, with R^2^ remaining at 42 and 43%, respectively.

A backward hierarchical multiple regression analysis using the same variables revealed that the BNIS Total score predicted higher level of discrepancy scores (accounting for 24% of the variability) with the number of invalid tapping responses in the non-dominant hand only marginally adding to the predictive power (now accounting for 26% of the variability). Delayed recall on the BVMT-R further added to the predictive power (now increasing the accounted variability to 30%). The BNIS Speech and Language subtest score, however, increased the amount of predicted variability by 13%, ultimately explaining 43% of the variance [*R*^2^ = 0.43, *F*(4, 26) = 4.84, *p* = 0.005].

## 4 Discussion

The findings of the present study support the hypothesis that patients with SMC tend to underreport their functional abilities and patients with MCI and early probable AD tend to overreport their functional abilities. This finding is compatible with other reports in the literature (25). The present study also provides supportive evidence for the hypothesis that older individuals with known or suspected memory impairments who overstate their functional abilities have lower test scores on neuropsychological measures sampling both cognitive and non-cognitive domains of functioning. This study is somewhat unique in that it included research participants that were expected to both overreport and underreport their functional abilities in comparison to relatives’ perceptions. This methodological approach was used to capture a larger range of disparate perceptions, thus providing a more detailed examination of the strength and direction of their neuropsychological correlates.

ISA has been associated with global cognitive impairment in patients with dementia (50). It is thus not surprising that overall measures of global cognitive functioning (such as the BNI Total Score and the WAIS-IV Full Scale IQ) had mild to moderate *negative* correlations with patients’ tendencies to overstate their functional abilities (*ρ* = −0.48 and *ρ* = −0.36, respectively). What is somewhat surprising and not an anticipated finding was that the BNI Speech and Language subtest score showed the highest correlation with overestimated functional abilities compared to relatives’ reports (*ρ* = −0.65). None of the research participants exhibited significant aphasia, as this would have precluded completion of the PCRS. The BNI Speech and Language subtest includes screening measures of auditory comprehension, naming, and the ability to add and subtract numbers. These latter abilities are known to decline in the early stages of a dementing condition (50). Patients with early AD showed the greatest tendency to overrate their functional competency (see Figure 1), which may be partly attributable to their difficulties comprehending the questions.

Not surprisingly, the level of memory impairment moderately correlated with the discrepancy scores of functional competencies (e.g., BVMT-R T-score, *ρ* = −0.54), as predicted. It is important, however, to note that the degree of the person’s memory impairment often does not correlate with the strength of their impaired awareness of their memory impairment (51).

Other factors than cognitive deficit per se may play a role in the apparent lack of awareness of functional limitations in everyday life. In this study, we explored whether motor and affective functions played additional roles. The number of invalid taps in the non-dominant hand *positively* correlated with discrepancy scores. The degree of correlation was moderate (*ρ* = 0.44). Invalid tapping responses may be a motor manifestation of a failure at executive control of sustained controlled movements when the individual is fatigued by the task [see (20)]. It may also reflect a breakdown in what Luria (52) referred to as the “kinetic melodies” of the brain. Regions underlying this ability were hypothesized to include frontal and parietal networks (52). In modern day terminology, these networks overlap with the Default Mode Network, which has been associated with frank anosognosia in persons with dementia (53), and within the posteromedial parietal cortex (PMC), which is affected by amyloid deposition in Alzheimer’s disease’s early stages (54). Ilardi et al. (55) hypothesized that evaluating PMC-related domains, such as episodic memory, hand-eye coordination, ISA, and working memory, could facilitate early diagnosis and have significant prognostic value for conversion from MCI to early AD.

In the present study, performance on the TMT Part B did not significantly correlate with discrepancy scores (*ρ* = −0.30, *p* = 0.08), indicating no strong association with either over- or underestimation. Additionally, the WAIS-IV composite score of working memory did not correlate with ISA (*ρ* = −0.29, *p* = 0.09). Other investigators have reported similar findings in individuals with MCI or mild dementia (12). While these findings do not support the hypothesis that impaired executive functioning is positively correlated with reduced self-awareness, it is possible that these measures of executive dysfunction are not sufficiently sensitive to predict such a relationship. For example, the number of preservative errors on the Wisconsin Card Sorting Test has been shown to be positively related to ISA in persons with severe TBI (56), while the number of categories achieved have not been (3). Determining exactly which measures of executive dysfunction may contribute to impaired self-awareness in MCI and early AD requires further investigation.

While the present study revealed a *positive* correlation between performance on the BNI Affect subtest with the relatives’ ratings of functional competencies in everyday life (*ρ* = 0.51, *p* = 0.003), the relationship of this measure of affect expression and perception to level of discrepancy scores revealed only a mild *negative* correlation (*ρ* = −0.36, *p* = 0.047). However, these associations were not statistically significant after more rigorous analysis. Connecting changes in affect expression and perception with impaired or reduced awareness of abilities versus disabilities is a complicated task. In people with SMC, they perceive themselves as less functionally competent than their relatives. This perception may relate to associated difficulties with anxiety and depression commonly reported in this heterogeneous group (57).

Depressive symptoms are known to negatively affect self-perception, and several studies have documented an inverse relationship between ISA and depression (17, 58, 59). In patients with SMC, preserved language abilities may allow for accurate comprehension of self-report items, but higher levels of depression might lead to a more pessimistic self-assessment and underreporting of abilities. Conversely, in patients with MCI and early probable AD dementia, subtle language deficits may interfere with accurate self-evaluation, potentially resulting in an overestimation of abilities despite lower depressive symptoms. Together, these findings suggest that depression could modulate self-report accuracy differently across clinical subgroups, warranting further investigation into the interplay between mood, language, and self-awareness in older adults.

It is important to note that the current study does not distinguish between ISA and denial in this patient population, a differentiation emphasized by Prigatano et al. (60). Both phenomena can contribute to discrepancies between self-reported and relative-reported functional abilities, yet their underlying mechanisms appear distinct. ISA is thought to represent a neuropsychological impairment, while denial appears to reflect a psychological method of reducing anxiety associated with cognitive decline (61). Future studies should incorporate structured clinical interviews and behavioral assessments that specifically probe for features of denial, such as avoidance of discussing distressing information or discouraging the examiner from asking further questions about the patient’s clinical state.

The major limitations of this study are the sample size and the potential bias of a convenience sample. Patients were originally recruited for this study because they were referred for a clinical neuropsychological evaluation. Several patients with SMC were not accompanied by a relative or significant other, and therefore, a PCRS-R was not obtained. The sample size was, therefore, relatively small. In contrast, many patients with early AD had trouble understanding the questions on the PCRS and could not complete it. Typically, only their relatives completed the PCRS-R. This resulted in a small number of AD patients having both a PCRS and PCRS-R data set.

We did not conduct a formal *a priori* power analysis, and sample size was limited to available participants. Accordingly, regression results should be interpreted cautiously and confirmed in future studies with larger samples. It is recognized that these convenience samples may not be representative of larger groups of patients with SMC, MCI, or early probable AD. However, these patients’ neuropsychological test findings provide supportive evidence that they demonstrate the type and level of cognitive impairments reported in the literature when large samples are studied (62, 63).

The absence of a formal measure of caregiver stress in our dataset may have influenced the informant ratings on the PCRS-R, potentially contributing to the observed group differences. Future research should address this by incorporating caregiver burden assessments to better understand its potential impact on informant-based evaluations.

In conclusion, we found that reduced self-awareness of functional abilities, measured by the discrepancy between patients’ and their relatives’ ratings, correlates with impairments in language, memory, affect perception and expression, finger tapping movements, and overall cognitive status. Notably, a tendency to overestimate functional abilities was linked to greater cognitive, affective, and motor deficits. These findings underscore that underlying language capabilities may influence how patients understand the questions being asked, potentially skewing findings on reduced awareness. Additionally, the present study highlights the importance of incorporating both cognitive and non-cognitive measures (i.e., affect and motor) when assessing factors associated with reduced or impaired self-awareness. Finally, the strong associations between relative reports of functional abilities and objective neuropsychological performance provides empirical support of the value of relatives’ independent ratings of functional competencies in older individuals with known or suspected memory impairments.

## Data Availability

The datasets presented in this article are not readily available because the datasets used in this study are not publicly available but can be made available from the corresponding author upon reasonable request. All in-house codes are available upon request. Requests to access the datasets should be directed to ashley.stokes@commonspirit.org.
